# Predictive Factors Affecting Long-Term Outcome of Unilateral Lateral Rectus Recession

**DOI:** 10.1371/journal.pone.0137687

**Published:** 2015-09-29

**Authors:** Hee Kyung Yang, Mi-Jin Kim, Jeong-Min Hwang

**Affiliations:** Department of Ophthalmology, Seoul National University College of Medicine, Seoul National University Bundang Hospital, Seongnam, South Korea; Boston Children's Hospital, UNITED STATES

## Abstract

**Background:**

There are few long-term outcome reports of unilateral lateral rectus (LR) recession for exotropia including a large number of subjects. Previous reports on unilateral LR recession commonly show extremely low rates of initial overcorrection and large exodrifts after surgery suggesting that the surgical dose may be increased. However, little is known of the long-term outcome of a large unilateral LR recession for exotropia.

**Objectives:**

To determine long-term outcomes and predictive factors of recurrence after a large unilateral LR recession in patients with exotropia.

**Data Extraction:**

Retrospective analysis was performed on 92 patients aged 3 to 17 years who underwent 10 mm unilateral LR recession for exotropia of ≤ 25 prism diopters (Δ) with prism and alternate cover testing and were followed up for more than 2 years after surgery. Final success rates within 10Δ of exophoria/tropia and 5Δ of esophoria/tropia at distance in the primary position, improvement in stereopsis and the predictive factors for recurrence were evaluated.

**Results:**

At 24 months after surgery, 54% of patients had ocular alignment meeting the defined criteria of success, 45% had recurrence and 1% had overcorrection. After a mean follow-up of 39 months, 36% showed success, 63% showed recurrence and 1% resulted in overcorrection. The average time of recurrence was 23.4±14.7 months (range, 1–60 months) and the rate of recurrence per person-year was 23% after unilateral LR recession. Predictive factors of recurrence were a larger preoperative near angle of deviation (>16Δ) and larger initial postoperative exodeviation (>5Δ) at distance.

**Conclusions:**

Long-term outcome of unilateral LR recession for exotropia showed low success rates with high recurrence, thus should be reserved for patients with a small preoperative near angle of exodeviation.

## Introduction

In patients with small to moderate angle exotropia, unilateral lateral rectus (LR) recession can save operation time as well as spare other rectus muscles for reoperation.[1–13] However, unilateral LR recession may result in undercorrections or recurrence and may cause incomitance.[1–13] The success rate of unilateral LR recession has been reported from 61% to 100%,[1–13] however, most of these studies have follow-up periods less than a year, performed variable amounts of recession, and used variable criteria of success. An initial overcorrection after exotropia surgery is generally considered desirable for a better long-term outcome.[14–18] However, regarding the extremely low rate of initial overcorrection after unilateral LR recession compared to unilateral recession and resection or bilateral LR recession,[13–17] the surgical dose of unilateral LR recession may need to be increased compared to previous studies. Therefore, in this study, a uniform maximal dosage of unilateral LR recession was performed,[19] and we determined the long-term success rates, together with predictive factors affecting surgical outcomes.

## Materials and Methods

A retrospective review of medical records was performed on 105 consecutive patients aged 3 to 17 years who underwent unilateral LR recession for exotropia of ≤ 25 prism diopters (Δ) at distance and near, between 2007 and 2009. The minimum required follow-up period after surgery was 24 months, except for patients who required reoperation for recurrence within 24 months. Patients with congenital anomalies, neurologic disorders, paralytic or restrictive strabismus, history of previous strabismus surgery, moderate to severe amblyopia, coexisting ocular diseases other than strabismus, and infantile exotropia were excluded. Patients with convergence insufficiency type exotropia, exodeviation greater at near than at distance of ≥10Δ, were excluded. Finally, a total of 92 consecutive patients were included. This study adhered to the Declaration of Helsinki and the protocol was approved by the Institutional Review Board of Seoul National University Bundang Hospital. All clinical investigation was conducted according to the principles expressed in the Declaration of Helsinki. Informed consent was not given, as patient records and information were anonymized and de-identified prior to analysis.

### Preoperative ophthalmologic examination

All patients underwent a complete ophthalmologic examination. We performed prism and alternate cover testing with accommodative targets for fixation at 1/3 and 6 m.

We performed patching in every patient who showed a distance-near deviation of >5Δ to exclude pseudodivergence excess-type exotropia. Refractive errors were determined by cycloplegic refraction and analyzed as spherical equivalent values. For patients with myopia of ≤ -1.00 diopter (D), spectacles of full cycloplegic refraction were prescribed. In patients with hyperopia of > +3.00D, spectacles of approximately +1.00 to +1.50D less than the full cycloplegic refraction were given. Spectacles were prescribed for patients with anisometropia of >1.50D between both eyes. In patients who needed glasses, preoperative measurements were made with correction. Amblyopia was defined as a difference of 2 lines or more between monocular visual acuities and only mild amblyopia with a difference of 2 lines were included. Lateral incomitance was defined as ≥5Δ change in the lateral gaze from the primary position. An A pattern was defined as an increase of 10Δ or more of exodeviation at downgaze compared with upgaze, and V pattern was defined as an increase of 15Δ or more of exodeviation at upgaze compared with downgaze. Sensory status was evaluated with the Randot stereoacuity test (StereoOptical Co, Inc. Chicago, IL, USA) at distance and near. Stereopsis of ≤100 seconds of arc was defined as good.

### Intraoperative procedures

Unilateral LR recession of 10 mm after the limbal incision was performed under general anesthesia by one surgeon (J-MH) as described.[20]

### Postoperative measurements

Postoperative assessments were made in the same manner at 1, 6, 12 months, and then every 1 year after the operation without or with correction in patients wearing glasses. Patients with postoperative esotropia were managed with full-time alternating patching. If the esotropia still existed at 1 month after the operation, hyperopia >+1.00D was corrected and base-out prisms were prescribed.[21,22]

Surgical outcome was considered successful if the alignment was between 10Δ of exophoria/tropia and 5Δ of esophoria/tropia at distance in the primary position. Recurrence was defined as an alignment of >10Δ of exophoria/tropia, and overcorrection defined as >5Δ of esophoria/tropia. Reoperation for recurrent exotropia was recommended in constant exotropia of ≥14Δ at distance, despite part-time occlusion or minus-lens therapy.

Improved stereopsis was defined as a decrease of 2 octaves or more, and decreased stereopsis was defined as an increase of 2 octaves or more at the last follow-up examination before reoperation.[23]

### Main outcome measures

Primary outcome measures were long-term surgical success based on postoperative alignment at distance and improvement in stereopsis. Secondary outcome measures were risk factors for recurrence after unilateral LR recession.

### Statistical analysis

Risk factors related to recurrence at 2 years after unilateral LR recession were evaluated by independent *t* test, Mann-Whitney U test and Pearson’s chi square test. Univariate and multivariate analyses by linear logistic regression were performed to identify significant risk factors affecting surgical outcomes, including preoperative distance and near angle of deviation, the difference of distance and near angle of deviation, age of onset, age at surgery, gender, constancy of deviation at distance and near, amblyopia, A and V pattern, lateral incomitance, stereopsis and initial postoperative angle of exodeviation at distance. Multivariate analyses revealed that only the initial postoperative angle of deviation at distance was significantly related to surgical outcomes (*P* = .001). Kaplan-Meier survival analysis was used to compare the cumulative probability of success between groups. A *P* value< .05 was considered as statistically significant and all analyses were performed with SPSS for Windows version 21.0 (SPSS Inc., Chicago, IL).

## Results

### Preoperative patient characteristics

The mean age of patients at surgery was 6.7 ± 2.2 years. The maximum preoperative angle of deviation was 19.0 ± 3.1Δ at distance and 16.5 ± 4.7 Δ at near.

### Surgical outcome

Immediately after surgery, the average change in postoperative alignment was 14.4±5.3 Δ at distance (range, 4–30Δ) and 13.3±5.4 Δ (range, 4–28Δ) at near. At 2 years after surgery, 50 of 92 (54%) patients had success, 41 patients (45%) had recurrence and 1 patient (1%) had overcorrection. After a mean follow-up of 39.1±18.0 months, 33 patients (36%) showed success, 58 patients (63%) showed recurrence and 1 patient (1%) showed overcorrection. The rate of exodrift from one month after surgery until the last follow-up was 3.0±3.8 Δ/year (range, -2.3–15.7Δ/year).

**Fig 1:**
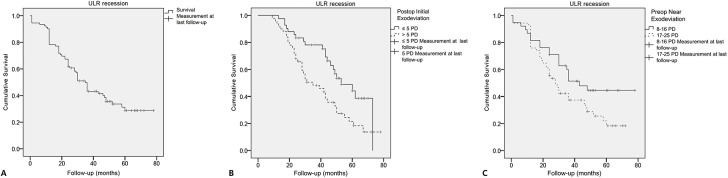
(A) Survival curve after unilateral lateral rectus recession for exotropia with the use of Kaplan-Meier survival analysis. (B) After stratification of the initial postoperative exodeviation at distance, the cumulative probability of success was higher in patients with a smaller initial postoperative exodeviation of 5Δ or less at distance (*P* = .008). (C) After stratification of the preoperative near angle of deviation, the cumulative probability of success was higher in patients with a smaller preoperative near angle of deviation of 16Δ or less (*P* = .049). Data were censored at the time of last follow-up examination.

In the 58 recurred patients, the average time to recurrence was 23.4±14.7 months (range, 1–60 months). The rate of recurrence per person-year was 23% after unilateral LR recession. The estimated mean time to recurrence was 41.1±3.0 months (Kaplan-Meier survival analysis, 95% confidence interval, 35.3–46.9 months) (Fig 1A). In these patients, the average rate of exodrift until 2 years after surgery was 6.0±3.7Δ/year (range, 1.9–15.7Δ/year).

Reoperation was performed in 49 patients (53%) after a mean interval of 33.4±15.9 months (range, 11–68 months) from the initial surgery.

### Predictive factors of recurrence

By univariate analysis, recurrence at 2 years after unilateral LR recession was significantly associated with a larger preoperative near angle of deviation (*P* = .044) and a larger initial postoperative angle of exodeviation at distance (*P* = .005) (Table 1). Other factors were not significantly related to recurrence, such as preoperative distance angle of deviation, the difference of distance and near angle of deviation, age of onset, age at surgery, gender, constancy of deviation at distance and near, amblyopia, A and V pattern, lateral incomitance, and stereopsis. Multivariate analyses revealed that only the initial postoperative angle of deviation at distance was significantly related to surgical outcomes (*P* = .001).

**Table 1:** Patient characteristics according to their surgical outcome at 2 years after unilateral lateral rectus muscle recession.

		Success (n = 50)	Recurrence (n = 41)	Overcorrection (n = 1)	*P* value
Age of onset (y)		5.5±2.6 (0~11)	5.7±3.1 (1~13)	4	.684^a^
Age at surgery (y)		6.7±2.2 (3~12)	6.7±2.3 (3~12)	4	.954^a^
Male Gender		22 (44%)	21 (51%)	1	.492^b^
Preoperative deviation	Distance (Δ)	18.6±3.2 (12~25)	19.6±2.7 (14~25)	20	.102^c^
	Near (Δ)	16.3±3.7 (8~25)	17.9±3.9 (8~25)	12	**.044** ^c^
	Distance- Near (Δ)	2.3±3.4 (-3~13)	1.7±3.5 (-7~10)	8	.664^c^
Initial postoperative deviation (Δ)^d^		4.0±4.4(-12~14)	6.6±3.8 (0~16)	-6	**.005** ^c^
Refractive error (D)		-0.30±2.28 (-7.1~+7.8)	-0.39±1.78 (-4.25~+3.38)	.88	.842^c^
Constant deviation	Distance	31 (62%)	25 (61%)	0	.980^b^
	Near	5 (10%)	9 (22%)	0	.270^b^
Amblyopia		11 (22%)	7 (17%)	0	.557^b^
Good Stereopsis (≤ 100 arcsec)		37/49 (76%)	25/37 (68%)	0	.416^b^
Recurrence (mo) (n=56)^e^		39.5±8.4 (28~58)	16.0±8.6 (1~30)		**<.001** ^c^
Last follow-up (mo)^f^		48.4±14.3 (24~78)	31.9±11.0 (19~69)	67	**<.001** ^c^

y = years; mo = months; Δ = prism diopters; D = diopters; *P* values were tested between groups of success and recurrence

^a^ *P* value by independent *t* test

^b^ *P* value by Pearson’s Chi square test

^c^ *P* value by Mann-Whitney U test

^d^A negative value for esodeviation and a positive value for exodeviation

^e^Time to recurrence of exotropia of >10Δ in 15 patients of the success group and 41 patients of the recurrence group

^f^Time to the last follow-up examination or until reoperation.

### Stratification of surgical outcomes

Surgical outcomes were stratified by significant predictive factors of recurrence; 1) initial postoperative angle of exodeviation at distance, and 2) preoperative angle of deviation at near. The preoperative angle of deviation at distance was not a significant predictive factor of recurrence.

#### Initial postoperative distance angle of deviation

Patients were subgrouped according to their initial postoperative distance angle of deviation at 1 month after surgery. The smaller group was defined as patients with an initial postoperative angle of ≤5Δ exodeviation, and the larger group with an angle of >5Δ exodeviation. The smaller group with an initial postoperative angle of ≤5Δ exodeviation had a significantly higher success rate at 2 years (67% vs 44%, *P* = .038) and at the final examination (48% vs 26%, *P* = .044).

The cumulative probability of success was higher in patients with a smaller initial postoperative angle of deviation (Kaplan-Meier survival analysis, *P* = .008, log rank test) (Fig 1B). In patients with a smaller initial postoperative angle of deviation (≤5Δ), the estimated mean time to recurrence was 52.8±3.4 months. In patients with a larger angle of deviation (>5Δ), the estimated mean time to recurrence was 40.0±3.2 months. The rates of recurrence per person-year after unilateral LR recession were 16% in the smaller group and 32% in the larger group.

#### Preoperative near angle of exodeviation

Patients were subgrouped according to their preoperative near angle of exodeviation. The smaller group was defined as patients with a near angle of exodeviation between 8 to 16Δ, and the larger group between 17 to 25Δ. The smaller group with a preoperative near angle of ≤16Δ exodeviation had a significantly higher success rate at 2 years (66% vs 46%, *P* = .045). However, final success rates were not significantly different (45% vs 30%, *P* = .112).

The cumulative probability of achieving successful ocular alignment was higher in patients with a smaller near angle of deviation (Kaplan-Meier survival analysis, *P* = .049, log rank test) (Fig 1C). In patients with a smaller preoperative near angle of deviation (8–16Δ), the estimated mean time to recurrence was 48.0±4.8 months. In patients with a larger preoperative near angle of deviation (17–25Δ), the estimated mean time to recurrence was 35.1±3.3 months. The rates of recurrence per person-year after unilateral LR recession were 17% in the smaller group and 28% in the larger group.

### Stereoacuity

Preoperatively, Stereoacuity could be tested in 87 patients, and good stereoacuity was present in 71% (62/87). At the last follow-up examination, stereoacuity testing could be performed on all subjects and good stereoacuity was present in 72% (66/92). Postoperative stereoacuity improvement was noted in 17 out of 87 patients (20%), 59 patients (68%) were stationary and 11 patients (13%) decreased by 2 or 3 octaves.

### Postoperative complication

There were no other postoperative complications, including symptomatic diplopia at lateral gazes or limitation in abduction or adduction in any patient throughout the follow-up period.

## Discussion

Our study showed that a large number of patients undergoing unilateral LR recession of 10 mm experience recurrence, particularly in those with an initial postoperative angle of >5Δ exodeviation at distance, or with an angle of >16Δ exodeviation at near. Therefore, it is very important to know the high risk of recurrence of this procedure. The poor long-term outcome of unilateral LR recession showing high recurrence rates suggest that this procedure should be reserved for patients with small angles of exotropia, especially at near. Our study should be remembered by strabismologists in deciding the type of surgery for small to moderate angles of exotropia.

Another study in 82 Korean patients with intermittent exotropia of 20–25Δ after unilateral LR recession achieved successful alignment ≤10Δ in 63% of patients at 2 years and 61% at the final follow-up, which was similar with our study up to 2 years (54%), but higher at the final follow-up (36%).[13] The reason of the relatively high rate of recurrence after 2 years in our study is not clear, but may be partly due to a possible selection bias towards patients with recurrence in a tertiary referral hospital. Kaplan-Meier survival analysis of the cumulative probability of achieving successful ocular alignment provides better comparison of surgical outcomes with different follow-up periods. Unfortunately, the former study did not provide this for comparison.[13]

The largest series of unilateral LR recession (7–10 mm) included 100 patients with intermittent exotropia of 15–35Δ at the age of 11 months to 15 years.[10] A successful alignment of an exodeviation of ≤5Δ and absence of any esotropia in the primary and lateral gaze at distance or near was achieved in 76% of patients at final follow-up of 0.5 to 7.4 years. There was only one overcorrected patient of 20Δ at 6 months. Sixteen patients (16%) underwent a second surgery for recurrent exotropia. The amount of initial postoperative exodeviation significantly correlated with final success rates, which is consistent with our study. The best result of unilateral LR recession was reported by Deutsch et al,[24] of which 100% achieved alignment ≤10Δ in 30 patients with 15–20Δ exotropia after 7.0 or 7.5 mm of unilateral LR recession, up to an average follow-up of 21 months.

To the best of our knowledge, the longest follow-up study of unilateral LR recession is 3 years.[6] Dadeya and Kamlesh[6] reported the success rate of 78% at 4 years (range, 3–8 years) after unilateral LR recession of 8 mm in 32 patients with intermittent exotropia of 25–30Δ. The mean amount of initial correction was 22.9Δ, which was larger than our study of 14.4Δ. They also found a continuous exodrift with time. In contrast, our study included patients with a smaller preoperative exodeviation of 19Δ (12–25Δ), and performed 10 mm of unilateral LR recession, which was larger than the previous studies. In the former study,[6] only 2 of 28 patients (7%) had poor fusional ability barely showing fusion with the Worth-4-dot test. In contrast, a high proportion of patients in our study had constant exotropia (62%) with poor fusional control, which may be partly responsible for the poor outcome. The small number of patients and including only basic type exotropia in the previous study may have also affected the results.

Regarding the predictive factors affecting the results of unilateral LR recession, we found that a larger initial postoperative angle of exodeviation at distance, and a larger preoperative angle of exodeviation at near are related to recurrence. Firstly, an initial overcorrection after exotropia surgery is generally considered desirable for a better long-term outcome.[14–18] Kim et al[13] found that an average exodrift of 3.4Δ (1.7Δ per year) occurred from one month after surgery until 2 years. In our study, the rate of exodrift from one month after surgery until the last follow-up was 3.0Δ per year, which was twice as large as the previous study.[13] Therefore, the continuous exodrift after surgery, as well as initial undercorrection, seems to be responsible for the large proportion of recurrence after unilateral LR recession. However, Dadeya and Kamlesh[6] reported that there was no significant difference in early vs late alignment after unilateral LR recession, and emphasized that early overcorrection is not desirable. Secondly, a larger preoperative angle of exodeviation at near was also related to recurrence, and although this was not proven by multivariate analysis, the cumulative probability of achieving successful ocular alignment was lower in this subgroup. The importance of this fact is that the preoperative near angle of exodeviation is also a point to be considered in addition to the distance angle when deciding to perform unilateral LR recession.

The major strength of unilateral LR recession would be the low rate of overcorrection, which could lower the risk of amblyopia or loss of stereopsis in young patients. However, if consecutive esotropia could be carefully managed with prism glasses, the risk of amblyopia or loss of stereopsis was minimal.[21,22] In our study, stereoacuity had improved or was stationary in most patients (87%) but postoperative decrease was found in 11 patients (14%). First of all, the repeatability of the stereotest is not good and can be variable even on the same day. Patients with decreased stereopsis showed a reduction of only 2 or 3 octaves, which is not a substantial amount. In addition, 9 out of the 11 patients with postoperative decrease showed recurrence at the final examination, thus deterioration of stereoacuity, if any, can be explained partly by recurrent exotropia.

The limitation of abduction after large lateral rectus recessions has not been clinically significant with unilateral LR recessions of 7–12 mm.[25] We also found no significant limitation of abduction after 10 mm of bilateral lateral rectus recession in large angle exotropia.[25] Deacon and colleagues [26] have reported that unilateral horizontal rectus muscle surgery may cause lateral incomitance and diplopia after 6 months of surgery in patients with exotropia. However, this study is different from ours because they combined the results of unilateral lateral rectus recession with that of unilateral lateral rectus recession and medial rectus resection, and not just ‘unilateral lateral rectus recession’ as in our study.[27] The lack of a short description of the surgical technique as well as a table of the surgical doses impedes our understanding of the surgical results.[27] In fact, addition of a resection of medial rectus muscle is believed to have a tethering effect and may increase the chance of overcorrection in the ipsilateral gaze.[27] Conversely, unilateral lateral rectus recession alone may not produce such incomitance. Livir-Rallatos et al.[28] even performed 15 mm of bilateral lateral rectus recession for exotropia of 60Δ, but did not encounter complications of abduction limitation.

This study has a few limitations. First, this study was retrospective, therefore the surgical indications and treatment options could not be randomized. Second, a maximum amount of 10 mm unilateral LR recession was performed on different amounts of preoperative deviations. This is the largest amount of unilateral LR recession compared to previous reports of 5–10 mm.[11] We performed a maximum recession under the hypothesis that the surgical dose of unilateral LR recession may be insufficient, regarding the extremely low rate of initial overcorrection after unilateral LR recession compared to other types of surgery, such as unilateral recession and resection or bilateral lateral rectus recession[13–17], and the larger postoperative exodrift after unilateral LR recession resulting in long-term recurrence.[13–18] However, the long-term rate of recurrence after a maximum amount of unilateral LR recession in this study was even higher compared to previous reports, implying that unilateral LR recession is inherent with a high risk of recurrence even with a maximum surgical dose. Therefore, a different type of surgery should be considered in cases with a high risk of recurrence. Lastly, convergence insufficiency type exotropia were not included, as they had mostly underwent unilateral recession and resection.[29] The previous study is consistent with our results, since patients with a larger preoperative near angle of deviation were more likely to recur, suggesting that unilateral recession and resection may be preferred in these patients to maintain a tethering effect against recurrence.[29]

In conclusion, in patients with small to moderate angles of exotropia, the long-term rate of successful ocular alignment was only 36% even after a large unilateral LR recession. Patients with an initial postoperative angle of exodeviation of >5Δ at distance, or a preoperative near angle of exodeviation of >16Δ had a higher risk of recurrence. The poor long-term outcome of unilateral LR recession showing high recurrence rates suggest that this procedure should be reserved for patients with small angles of exotropia, especially at near.
